# Optimal control of monomers and oligomers degradation in an Alzheimer’s disease model

**DOI:** 10.1007/s00285-025-02256-3

**Published:** 2025-08-08

**Authors:** Iulia Martina Bulai, Francesco Ferraresso, Francesca Gladiali

**Affiliations:** 1 Department of Chemical, Physical, Mathematical and Natural Sciences, Via Vienna 2, 07100 Sassari, Italy; 2 Department of Mathematics “Giuseppe Peano”, University of Torino, via Carlo Alberto 10, 10123 Torino, Italy; 3 Department of Computer Science, Strada Le Grazie 15, Ca’ Vignal 2, 37134 Verona, Italy

**Keywords:** Alzheimer disease, Optimal control, ODEs, Monomer degradation, Oligomer degradation, 92B, 92-10, 92C50, 37N25, 34C60, 34H05

## Abstract

The aggregation and accumulation of oligomers of misfolded A$\beta$-amyloids in the human brain is one of the possible causes for the onset of the Alzheimer’s disease in the early stage. We introduce and study a new ODE model for the evolution of Alzheimer’s disease based on the interaction between monomers, proto-oligomers, and oligomers of A$\beta$ amyloid protein in a small portion of the human brain, based upon biochemical processes such as polymerization, depolymerization, fragmentation and concatenation. We further introduce the possibility of controlling the evolution of the system via a treatment that targets the monomers and/or the oligomers. We observe that a combined optimal treatment on both monomers and oligomers induces a substantial decrease of the oligomer concentration at the final stage. A single treatment on oligomers performs better than a single treatment on monomers. These results shed a light on the effectiveness of immunotherapy using anti-A$\beta$ antibodies, targeting monomers or oligomers. Several numerical simulations show how the oligomer concentration evolves without treatment, with single monomer/oligomer treatment, or with a combined treatment.

## Introduction

Alzheimer’s disease (AD) is one of the most common neurodegenerative disorder, affecting millions of people worldwide and severely worsening their quality of life. It is characterized by progressive cognitive decline, loss of memory, synaptic dysfunction, and neuronal loss, WHO (2023). One of the main recognisable pathological effects of AD is the presence of amyloid-beta (A$\beta$) plaques and neurofibrillary tangles composed of tau protein in the brain of AD patients. Among the various hypotheses proposed to explain the etiology of AD, the Amyloid Cascade Hypothesis (ACH) remains one of the most widely studied and debated, Hardy and Higgins (1992).

The ACH states that the misfolding and aggregation of A$\beta$ peptides, particularly A$\beta _{42}$, act as a primary trigger in the disease progression, leading to synaptic impairment, tau pathology, neuroinflammation, and ultimately neuronal death, Chiti and Dobson (2017). A$\beta$ aggregation follows a complex nucleation-dependent polymerization process, starting with monomer misfolding and oligomer formation, followed by protofibril and fibril development, ultimately leading to mature amyloid plaques, Kamatham et al. (2024). Under physiological conditions, A$\beta$ exists in a dynamic equilibrium between monomeric, oligomeric, and fibrillar forms. However, in AD, an imbalance in A$\beta$ production, clearance, or aggregation, results in the formation of toxic oligomers and fibrils that disrupt neuronal function.

Emerging evidence, Kamatham et al. (2024), suggests that A$\beta$ oligomers, rather than mature fibrils, are the most neurotoxic species, capable of inducing oxidative stress, mitochondrial dysfunction, and synaptic loss. Despite extensive research, the precise mechanisms linking A$\beta$ misfolding to AD progression remain incompletely understood. Therapeutic strategies targeting A$\beta$ have recently started to yield positive results, confirming the pivotal role of A$\beta$ amyloids in the progression of AD, Aisen (2005); Sancesario et al. (2018); in the USA, two anti-amyloid monoclonal antibodies have been recently approved for the treatment of AD, after showing slowing of clinical decline in the trials, Aisen (2005); Cummings et al. (2021).

In Tamagno et al. (2018) an in vitro study in cerebrospinal fluid of Alzheimer’s disease patients was done in order to analyze the degradation efficacy of bromelain on monomers and oligomers. Acting on monomers and oligomers before the amyloid plaques are formed is important for preventing or delaying cognitive decline, Härd and Lendel (2012). In particular, it was shown that A$\beta$ monomers has an important role in sustaining the pathogenesis of AD, Tamagno et al. (2018), and, beside acting on the oligomers, Kass et al. (2022), also the removal of monomers might help for developing new therapeutic approaches to treat the disease.

In addition to in vitro and in vivo experiments for therapeutic drug development of Alzheimer’s disease also the mathematical models play an important role, Andrade-Restrepo et al. (2021); Bertsch et al. (2018, 2016); Andrade-Restrepo et al. (2020); Hao and Friedman (2016); Brennan and Goriely (2025); Ficiarà et al. (2023); Ramos et al. (2021); Menale and Travaglini (2024). In fact, the models, can be used as non invasive tools to better understand the disease and the interaction between the main actors of it. Moreover, by using optimal control theory, Hao et al. (2022); Torres et al. (2025); Rabiei et al. (2025), one can study the effect of the treatments in time on the populations of the models.

In this paper a novel mathematical model for the biochemical interactions between monomers, proto-oligomers, and oligomers of A$\beta$ amyloids, is introduced, Ashe (2020). The interactions between the populations of the ordinary differential equation (ODE) model are: polymerization, depolymerization, fragmentation, degradation and concatenation. Differently than in literature, here, we have also considered the concatenation terms between proto-oligomers of length $> 1$, as was done in Andrade-Restrepo et al. (2021) for the Partial Differential Equation (PDE) model. More importantly, here we have introduced two control terms, one for the degradation of the monomers and a second one for the degradation of the oligomers. Four different optimal control formulations were introduced assuming only the monomers or oligomers are treated, as well as both of them, with different treatments or the same treatment, respectively. Numerical results for all the cases complete the study, on one side by comparing the effect of different treatments on the monomers and oligomers and, on the other side, by analyzing the role of some key parameter values and of the initial conditions on the output of the optimal control formulations.

In the next section the ODE system is introduced. In Sect. 3 some analytical facts related to the boundedness of the solution and the equilibrium points of the model are reported. In Sect. 4, four different optimal control formulations are studied, i.e. CM, CO, CMO, CMOST, related to the control of the monomers, oligomers, both monomers and oligomers with different treatments and the same treatment, respectively. In Sect. 5 numerical results related to the optimal control formulations introduced in Sect. 4 are compared. Finally, Sect. 6 contains a summary of the main results of the paper.

## Model formulation

In this paper we introduce a five dimensional ODE system, whose populations are: the concentration of monomers, denoted by *M*, the concentration of proto-oligomers of length $l=2$, 3 or 4, denoted by $U_2$, $U_3$, $U_4$, respectively and the concentration of oligomers of length $l \ge 5$, denoted by *O*. The concentrations of monomers, proto-oligomers, and oligomers are assumed to interact according to the following biochemical processes:

1. Polymerization. A monomer and a proto-oligomer of length $l=2$, 3 or 4 react together to produce a proto-oligomer of length $l+1$, with rate $r_{l}$.
2. Depolymerization. A proto-oligomer of length $l=3$ or 4 breaks down due to oxydation or flaws in the chain, losing a monomer from the bottom or the top of the chain, with rate *b*.
3. Fragmentation. A proto-oligomer of length $l=2$, 3 or 4 breaks down into monomers or shorter proto-oligomers, with rate $\beta (l-1)$ depending on the length *l* of the chain. The coefficients $l-1$ were introduced in Doumic et al. (2009) under the assumption that the probability of obtaining a proto-oligomer of length *i* from one of length *l* is uniform. In other words, we are assuming that each of the $l-1$ bonds in proto-oligomer of length *l* has the same probability of breaking, thus implying that the fragmentation rate is linear in the proto-oligomer length.
4. Degradation. Monomers undergo proteolytic degradation due to peptidases and proteinases, with rate of concentration reduction $\delta$.
5. Concatenation. The aggregation between two proto-oligomers of length $j=2$, 3 or 4 and $k=2$, 3 or 4, produce a proto-oligomer of length $j+k$, with rate $a_{jk}$ and the aggregation between two monomers of length 1, produce a proto-oligomer of length 2 with rate $a_{11}$.

The underlying assumption is that proto-oligomers of length $l \ge 5$ are considered oligomers, which do not further interact with monomers or proto-oligomers. The choice of the length $l_o=5$ as the threshold for oligomer transformation is mainly assumed to simplify the analysis, whereas in the biochemical literature $l_o = 20$ or $l_o = 50$ is usually chosen. We notice however that simulations with a higher threshold are qualitatively very similar. In the standing assumptions, a natural description of the time evolution of the concentrations of monomers, proto-oligomers, and oligomers is given by the following non-linear, fully-coupled, system of ODEs:

1 $$\begin{aligned} \dfrac{dM}{dt}= & s M \left( 1-\dfrac{M}{K}\right) - M (r_2 U_2+r_3 U_3+r_4 U_4)+(b+2 \beta )U_3 + (b+2 \beta ) U_4 + 2 \beta U_2- a_{11} M^2+ \nonumber \\ & - \delta M- \alpha _0 v(t) M, \nonumber \\ \dfrac{dU_2}{dt}= & -r_2 MU_2 - \beta U_2 + (b+2 \beta )U_3+ 2 \beta U_4 - a_{23} U_2 U_3- a_{24} U_2 U_4 -a_{22} U_2^2 + a_{11} M^2,\nonumber \\ \dfrac{dU_3}{dt}= & -M (r_3 U_3 -r_2 U_2) - (2 \beta +b) U_3 +(b+ 2 \beta ) U_4 -a_{23}U_2 U_3 -a_{34} U_3 U_4- a_{33} U_3^2, \nonumber \\ \dfrac{dU_4}{dt}= & -M(r_4 U_4-r_3 U_3)- (b+3 \beta )U_4 - a_{24} U_2 U_4 -a_{34} U_3 U_4-a_{44}U_4^2 + a_{22}U_2^2, \nonumber \\ \dfrac{dO}{dt}= & r_4 MU_4 +a_{23} U_2 U_3 +a_{24}U_2 U_4+a_{34} U_3 U_4 +a_{33}U_3^2 +a_{44} U_4^2 -mO^2- \alpha _1 u(t)O, \end{aligned}$$

with initial conditions $M(t=0) = M_0$, $U_2(t=0) = U_{20}$, $U_3(t=0) = U_{30}$, $U_4(t=0) = U_{40}$, $O(t=0) = O_{0}$.

All the parameters assume non negative values and their meaning is the following: $r_i$, $i=2,3,4$ is the polymerization rate; *b* is the depolymerization rate; $\beta$ is the fragmentation rate; $\delta$ is the natural degradation rate of the monomers; while $a_{jk}$, with $j=1,2,3,$ or 4 and $k=1,2,3,$ or 4, is the concatenation rate. Here we also assume that the concentration of the monomers follow a logistic growth law (Whittington et al. 2018; Hao et al. 2022), with intrinsic growth rate *s* and carrying capacity *K*, while the population of the oligomers experiences “intraspecific competition” for space at rate *m*. Moreover, two different type of treatment can be introduced, a first one that act on the monomers population and degrade them at rate *v*(*t*), Tamagno et al. (2018); Sancesario et al. (2018), and a second one that act on the oligomers population, degrading it at rate *u*(*t*), Kass et al. (2022); Sancesario et al. (2018). In model (1) $\alpha _0$ and $\alpha _1$ can assume the value 0 or 1, depending if the treatment of only monomers, only oligomers or both treatments are considered.

In Table 1 are summarized the parameters related to model (1), next we will also introduce the values used for the simulations.

**Table 1:** Parameters of model (1). $1M = 1 l \ (mol)^{-1}$, is the molar concentration unit

Parameter	Name	Unit
*s*	Monomers intrinsic growth rate	$\hbox {s}^{-1}$
*K*	Monomers carrying capacity	$\hbox {M}^{-1}$
$r_i$, $i=2,3,4$	Polymerization rate	M^−1^ s^−1^
*b*	Depolymerization rate	$\hbox {s}^{-1}$
$\beta$	Fragmentation rate	$\hbox {s}^{-1}$
$\delta$	Degradation rate of monomers	$\hbox {s}^{-1}$
$a_{11}$	Concatenation rate	M^−1^ s^−1^
$a_{22}$	Concatenation rate	M^−1^ s^−1^
$a_{23}$	Concatenation rate	M^−1^ s^−1^
$a_{24}$	Concatenation rate	M^−1^ s^−1^
$a_{33}$	Concatenation rate	M^−1^ s^−1^
$a_{34}$	Concatenation rate	M^−1^ s^−1^
$a_{44}$	Concatenation rate	M^−1^ s^−1^
*m*	Oligomers intraspecific competition rate	M^−1^ s^−1^

## Some analytic facts

Let us assume throughout this section that $\alpha _0 = \alpha _1 = 0$, this simplification is needed in order to study the model (1) without control and to perform a linear stability analysis of it. We first note that the unique solution to (1) remains bounded for all times, with an explicit upper bound.

### Lemma 3.1

Given an initial datum $\Phi _0 = (M_0, U_{20}, U_{30}, U_{40}, O_0)$, with non-negative components, the unique non-negative solution $\Phi = (M, U_2, U_3, U_4, O)$ of system (1) is bounded for all times.

### Proof

By rewriting (1) as

$$\begin{aligned} \dfrac{d\Phi }{dt} = F(\Phi ), \end{aligned}$$

one notes that *F* is a locally Lipschitz function from $\mathbb {R}^5$ to $\mathbb {R}^5$. The classical Cauchy-Banach theory applies and yields the existence and uniqueness of a local classical solution. Let us set $T> 0$ to be the maximal time of existence of the solution with initial datum $\Phi _0$. By considering the first equation separately, and by comparing the solution *M* with 0, one concludes that $M \ge 0$, and similarly it must be that all the components of the local solutions are non-negative (and strictly positive if the initial datum is so). Let us define $f(t) = ((M(t) - q_1)^+)^2$, where $q_1 > M_0$ is a constant to be determined. Note that $f(0) = 0$. Moreover,

$$\begin{aligned} \begin{aligned}&f'(t) = 2 (M(t) - q_1)^+ \dfrac{dM}{dt} \\&= 2(M(t) - q_1)^+ \left[ s M \bigg (1-\dfrac{M}{K}\bigg ) - M (r_2 U_2+r_3 U_3+r_4 U_4 + \delta ) \right. \\ &\quad \left. +(b+2 \beta )(U_3+U_4) + 2 \beta U_2- a_{11} M^2 \right] . \end{aligned} \end{aligned}$$

It is not difficult to check that if

$$\begin{aligned} q_1 = \max \bigg \{ M_0, \frac{b + 2\beta }{r_3}, \frac{b + 2\beta }{r_4}, \frac{2\beta }{r_2}, K \chi _{\{s-\delta > 0\}} \bigg \}, \end{aligned}$$

then $f'(t) \le 0$ for all $t \in [0, T)$. Since $f(0) = 0$, and $f(t) \ge 0$ for all $t \in [0, T)$, it must be $f = 0$, and therefore $0 \le M(t) \le q_1$ for all $t \in [0, T)$. Repeating this reasoning in the other equations we obtain that $0 \le U_j(t) \le q_j$, where

$$\begin{aligned} q_2= & \max \bigg \{ U_{20}, \frac{b + 2\beta }{a_{23}}, \frac{2\beta }{a_{24}}, \frac{a_{11}q_1}{r_2} \bigg \},\\ q_3= & \max \bigg \{U_{30}, \frac{b + 2\beta }{a_{34}}, \frac{r_2 q_1 q_2}{2 \beta + b + q_1 r_3 + a_{23} q_2}\bigg \},\\ q_4= & \max \bigg \{U_{40}, \frac{b + 2\beta }{a_{34}}, \frac{q_1q_3 r_3 + a_{22}q_2^2}{q_1 r_4 + b + 3\beta + a_{24}q_2 + a_{34}q_3}\bigg \},\\ q_5= & \max \bigg \{O_0, \frac{r_4 q_1 q_4 + a_{23} q_2 q_3 + a_{24} q_2 q_4 + a_{34} q_3 q_4 + a_{33} q_3^2 + a_{44} q_4^4}{m}\bigg \}. \end{aligned}$$

We conclude that $\sup _{t \in [0, T)}|\Phi (t)| < + \infty$; but since the upper bounds $q_j$ do not depend on *t*, $\Phi (t)$ remains bounded as $t \rightarrow T^-$. We conclude that it must be $T = + \infty$, and $\sup _{t \in [0, + \infty )} |\Phi (t)| < \infty$. $\square$

In general system (1) does not admit explicitly computable solutions. We can however establish some semi-explicit bounds as follows.

### Lemma 3.2

Let $O(0):= O_0 > 0$. Then the last component *O*(*t*) of the solution to (1) admits the bounds

2 $$\begin{aligned} \frac{O_0}{1 + m t O_0 } \le O(t) \le \frac{(Q + O_0 \sqrt{mQ}) e^{2 \sqrt{mQ} t} - Q + \sqrt{mQ} O_0}{(\sqrt{mQ} + m O_0)e^{2 \sqrt{m Q} t} - O_0 m + \sqrt{m Q}}, \end{aligned}$$

with

$$\begin{aligned} Q = \max _{t \in [0, + \infty )} (r_4 MU_4 +a_{23} U_2 U_3 +a_{24}U_2 U_4+a_{34} U_3 U_4 +a_{33}U_3^2 +a_{44} U_4^2)(t). \end{aligned}$$

Given an initial datum $(M_0,0,0,0,0)$, with $M_0$ small, the first component *M*(*t*) of the solution to (1) is increasing for $s - \delta \ge 0$, decreasing for $s- \delta < 0$, for small values of $t>0$.

### Proof

From the last equation of (1) we deduce that

$$\begin{aligned} \dfrac{dO}{dt}+ m O^2 = r_4 MU_4 +a_{23} U_2 U_3 +a_{24}U_2 U_4+a_{34} U_3 U_4 +a_{33}U_3^2 +a_{44} U_4^2 \ge 0. \end{aligned}$$

The comparison principle implies that the solution *O*(*t*) is always greater or equal than $\tilde{O}(t)$, where

$$\begin{aligned} \dfrac{d\tilde{O}}{dt}= - m \tilde{O}^2, \qquad \tilde{O}(0) = O_0. \end{aligned}$$

An explicit computation shows that $\tilde{O}(t) = \frac{O_0}{1 + m t O_0 }$. Similarly, one sees that $O(t) \le \hat{O}(t)$ for all *t*, where

$$\begin{aligned} \hat{O}'(t) = Q - m \hat{O}^2(t), \qquad \hat{O}(0) = O_0. \end{aligned}$$

An explicit computation shows that

$$\begin{aligned} \hat{O}(t) = \frac{(Q + O_0 \sqrt{mQ}) e^{2 \sqrt{mQ} t} - Q + \sqrt{mQ} O_0}{(\sqrt{mQ} + m O_0)e^{2 \sqrt{m Q} t} - O_0 m + \sqrt{m Q}}. \end{aligned}$$

The second claim follows by observing that for $M_0 \le \varepsilon$, $((s- \delta ) - (a_{11} + s/K) C \varepsilon ) M \le \dfrac{dM}{dt} \le (s-\delta ) M$, for a suitable constant $C>0$ depending on the modulus of continuity of *M*; the solution is therefore very close to $M_0e^{(s-\delta )t}$, for $t \in [0, t_0]$. $\square$

We now proceed to analyse the equilibrium points and their stability. The equilibrium equations are given by (1) with $\alpha _0=\alpha _1=0$ and with 0 at the l.h.s. The last equation gives the unique positive solution

3 $$\begin{aligned} O=\sqrt{\frac{1}{m}\left( r_4 MU_4 +a_{23} U_2 U_3 +a_{24}U_2 U_4+a_{34} U_3 U_4 +a_{33}U_3^2 +a_{44} U_4^2\right) .} \end{aligned}$$

**Fig. 1:**
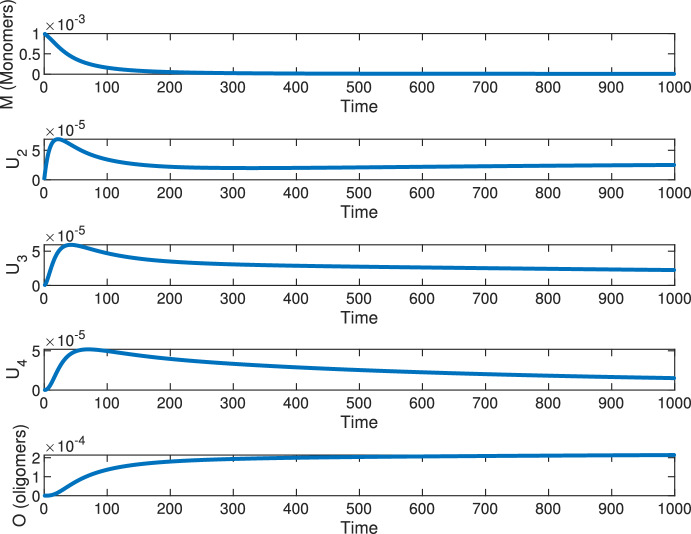
The state variables of model (1) with $\alpha _0=\alpha _1=0$ converging to the coexistence of the five populations

It is not difficult to check that either $O = 0$, corresponding to the trivial equilibrium $\textbf{0}=(0,0,0,0,0)$; or $O > 0$, in which case $M > 0$ and $U_j > 0$ for all $j = 2,3,4$. Therefore all the other possible equilibria are in the form $\textbf{E} = (M^*, U_{2}^*, U_3^*, U_4^*, O^*)$, with all positive components. See Fig. 1 for one possible coexistence equilibrium obtained numerically for a chosen set of parameter values and initial conditions.

$$\bullet$$ *The equilibrium* $$\textbf{0}=(0,0,0,0,0)$$.

The Jacobian of the model is the matrix

$$\begin{aligned} \textbf{J} = \begin{bmatrix} j_{11}& -r_2M+2\beta & -r_3M+b+2\beta & -r_4M+b+2\beta & 0 \\ -r_2U_2+2a_{11}M & j_{22} & b+2\beta -a_{23}U_2 & 2\beta -a_{24}U_2& 0 \\ -r_3U_3+r_2U_2 & r_2M-a_{23}U_3 & j_{33}& b+2\beta -a_{34}U_3& 0\\ -r_4U_4+r_3U_3 & -a_{24}U_4+2a_{22}U_2 & r_3M-a_{34}U_4 & j_{44}& 0\\ r_4U_4 & a_{23}U_3+a_{24}U_4 & a_{23}U_2+a_{34}U_4+2a_{33}U_3 & j_{54} & -2mO \end{bmatrix}\,, \end{aligned}$$

where

$$\begin{aligned} j_{11}= & s \left( 1-\dfrac{2M}{K}\right) - (r_2 U_2+r_3 U_3+r_4 U_4)- 2 a_{11} M - \delta , \\ j_{22}= & - r_2 M-\beta -a_{23}U_3-a_{24}U_4-2a_{22}U_2, \\ j_{33}= & - r_3 M-b-2\beta -a_{23}U_2-a_{34}U_4-2a_{33}U_3, \\ j_{44}= & - r_4 M-b-3\beta -a_{24}U_2-a_{34}U_3-2a_{44}U_4, \\ j_{54}= & r_4M+a_{24}U_2+a_{34}U_3+2a_{44}U_4. \end{aligned}$$

At the equilibrium $\textbf{0}$ then

$$\begin{aligned} \textbf{J} _\textbf{0}= \begin{bmatrix} s-\delta & 2\beta & b+2\beta & b+2\beta & 0 \\ 0 & -\beta & b+2\beta & 2\beta & 0 \\ 0& 0 & -b-2\beta & b+2\beta & 0\\ 0 & 0 & 0 & -b-3\beta & 0\\ 0 & 0 & 0 & 0 & 0 \end{bmatrix}\,. \end{aligned}$$

The eigenvalues of $\textbf{J} _\textbf{0}$ are

$$\begin{aligned}\lambda _1=s-\delta \, \ \lambda _2=-\beta \, \ \lambda _3=-b-2\beta \, \ \lambda _4=-b-3\beta \, \ \lambda _5=0. \end{aligned}$$

When $s-\delta > 0$, the equilibrium **0** is unstable since $\textbf{J} _\textbf{0}$ has eigenvalues of opposite sign. When $s-\delta < 0$ instead, we cannot conclude anything from the analysis of the linearised system. We note however that if the initial datum is in the form (0, 0, 0, 0, *a*), $a > 0$, then the solution (0, 0, 0, 0, *O*(*t*)) to system (1) is explicitly computable and it is given by

$$\begin{aligned} O(t) = \frac{a}{1 + m t a }. \end{aligned}$$

Thus, we see that for this particular choice of initial data the solution is stable and tends asymptotically to the equilibrium $\textbf{0}$.

## Optimal control formulation

In this section we focus on some optimal control problems related to (1). The state variables of the system are the populations *M*(*t*), $U_2(t)$, $U_3(t)$, $U_4(t)$ and *O*(*t*) in (1) while the two controls *v*(*t*) and *u*(*t*) can be seen as two treatments that degrade the monomers’s population *M*(*t*) and the oligomer’s population *O*(*t*), respectively. Our goal is to minimize a suitable objective functional, denoted by *J*, that considers the populations of monomers *M*(*t*) and oligomers *O*(*t*) over time, the final populations at the treatment’s conclusion ($t = T$), as well as the costs of the controls, their interaction, and potential side effects over time. To provide a comprehensive overview of the possible scenarios, we address various cases of optimal control here. We will then compare the results obtained in Sects. 5 and 6. In each case, we will introduce an appropriate objective functional *J*, which will be defined accordingly. The first case involves two treatments, *v*(*t*) and *u*(*t*), acting separately on the populations of monomers *M*(*t*) and oligomers *O*(*t*), respectively, called CMO from now on. In the second and third cases, we choose to apply a single treatment, either *v*(*t*) or *u*(*t*), targeting their respective populations, called CM and CO from now on, respectively. Finally, in the last case, we consider a single treatment acting simultaneously on both populations, called CMOST.

### Optimal control acting on both monomers and oligomers (CMO)

In this subsection we will consider the controls *v*(*t*) and *u*(*t*) which act separately on the monomers’s population *M*(*t*) and the oligomer’s population *O*(*t*), respectively, in the model (1) with $\alpha _0=\alpha _1=1$. We will introduce a given objective functional in order to minimize both populations *M*(*t*) and *O*(*t*) at the end of the treatment ($t=T$ with weights $c_1, c_2$) and over the treatment (in [0, *T*] with weights $c_3, c_4$), the cost of the two therapies over the treatment (with weights $c_5, c_7$), their side effects over the treatment (with weights $c_6, c_8$) and their possible interactions (with weight $c_9$), i.e.

4 $$\begin{aligned} J(v,u)= & c_1 M(T) +c_2 O(T)+ \int _0^T \left[ c_3 M(t)+c_4 O(t) +c_5 v(t) \right. \nonumber \\ & \left. +c_6 v(t)^2 +c_7u(t)+c_8u(t)^2+c_9v(t)u(t)\right] dt, \end{aligned}$$

where $c_i$, with $i=1,2,...,9$, are positive constants that we choose generically so that they can be adapted to the needs of the case, that must satisfy the sole condition $4c_6c_8-c_9^2>0$. This last assumption ensures the convexity of the objective functional with respect to the controls and means that the interaction between the two treatments should not outweigh the side effects of *u* and *v* too much. In fact, the case $c_6 = c_8 = c_9$ is admissible. The state variables *M*(*t*), $U_2(t)$, $U_3(t)$, $U_4(t)$ and *O*(*t*) and the controls *v*(*t*) and *u*(*t*) are subject to (1) and $M(t=0) = M_0$, $U_2(t=0) = U_{20}$, $U_3(t=0) = U_{30}$, $U_4(t=0) = U_{40}$, $O(t=0) = O_{0}$. The initial conditions $M_0, U_{20},U_{30},U_{40},O_{0}$, can be seen as measurements of the concentrations of structured A$\beta$-amyloids in the brain, and can be targeted to a specific individual. Our aim is to seek an optimal control $(v^*,u^*)$ such that

5 $$\begin{aligned} J(v^*,u^*) = \min _{(v,u) \in W} J(v,u), \end{aligned}$$

where $$W= \lbrace (v(t), u(t)) \in L^{\infty }( \left[ 0,T \right] )\times L^{\infty }( \left[ 0,T \right] )$$$$\quad | \quad 0 \le v(t) \le v_{max},$$$$\quad 0 \le u(t) \le u_{max} \rbrace$$. $$v_{max}$$, $$u_{max}$$ are the maximum rate of the therapies *v* and *u* respectively.

Since the controls *v* and *u* and the state variables are uniformly bounded in $L^\infty$ and the problem is convex in the controls, then standard control theory, see Lenhart and Workman (2007), can be used to prove the existence of an optimal control $(v^*,u^*)$. In order to find it, by standard control theory, we introduce five adjoint variables, respectively $\lambda _i$, $i=1,2,...5$, one for each state variable and form the Hamiltonian:

6 $$\begin{aligned} H= & c_3 M(t)+c_4 O(t) +c_5 v(t)+c_6 v(t)^2 +c_7u(t)+c_8u(t)^2+c_9v(t)u(t)+ \lambda _1 {\dfrac{dM}{dt}}+ \lambda _2 \dfrac{dU_2}{dt} \nonumber \\ & + \lambda _3 \dfrac{dU_3}{dt}+ \lambda _4 \dfrac{dU_4}{dt}+ \lambda _5\dfrac{dO}{dt}, \end{aligned}$$

where $\dfrac{dM}{dt}$, $\dfrac{dU_2}{dt}$, $\dfrac{dU_3}{dt}$, $\dfrac{dU_4}{dt}$ and $\dfrac{dO}{dt}$ are the RHS of the ODE system (1) with $\alpha _0=\alpha _1=1$. By the Pontryagin’s Maximum Principle, the adjoint ODE system reads

7 $$\begin{aligned} \dfrac{d \lambda _1}{dt}= & - \dfrac{\partial H}{ \partial M} = - \biggl [ c_3 + \lambda _1 (s- \delta -v(t)) + M \left[ \dfrac{-2 \lambda _1 s}{K} -2 a_{11} \lambda _1 + 2 a_{11} \lambda _2\right] + \biggr . \nonumber \\ & \biggl . + r_2 U_2(- \lambda _1 - \lambda _2 + \lambda _3) + r_3 U_3 (- \lambda _1 - \lambda _3+ \lambda _4) +r_4 U_4 (-\lambda _1 - \lambda _4 + \lambda _5) \biggr ], \nonumber \\ \dfrac{d \lambda _2}{dt}= & - \dfrac{\partial H}{ \partial U_2} = - \left[ \beta (- \lambda _2 + 2 \lambda _1) + r_2 M (- \lambda _1 - \lambda _2 + \lambda _3) + 2 a_{22} U_2 (-\lambda _2 + \lambda _4)+ \right. \nonumber \\ & \left. +a_{23} U_3 (- \lambda _2 - \lambda _3 + \lambda _5) + a_{24} U_4 (- \lambda _2 - \lambda _4 + \lambda _5) \right] , \nonumber \\ \dfrac{d \lambda _3}{dt}= & - \dfrac{\partial H}{ \partial U_3} = - \left[ (b+2 \beta ) (\lambda _1 + \lambda _2 - \lambda _3) +r_3 M (- \lambda _1 - \lambda _3 + \lambda _4)+ \right. \nonumber \\ & \left. +a_{23} U_2 (- \lambda _2 - \lambda _3 + \lambda _5) +2 a_{33} U_3 (-\lambda _3+ \lambda _5) +a_{34} U_4 (- \lambda _3 - \lambda _4 + \lambda _5) \right] , \nonumber \\ \dfrac{d \lambda _4}{dt}= & - \dfrac{\partial H}{ \partial U_4} = -\left[ (b+2 \beta ) (\lambda _1 + \lambda _3) + 2 \beta \lambda _2 -(b+3 \beta ) \lambda _4+ r_4 M (-\lambda _1 - \lambda _4 + \lambda _5)+ \right. \nonumber \\ & \left. +a_{24} U_2 (-\lambda _2 - \lambda _4 + \lambda _5) +a_{34} U_3 (-\lambda _3 - \lambda _4 + \lambda _5) +2 a_{44} U_4 (-\lambda _4+ \lambda _5) \right] , \nonumber \\ \dfrac{d \lambda _5}{dt}= & - \dfrac{\partial H}{ \partial O} = -(c_4 + \lambda _5 (- 2 M O - u(t)) ), \end{aligned}$$

with the transversality conditions $\lambda _1(T)=c_1$, $\lambda _2(T)=\lambda _3(T)=\lambda _4(T)=0$ and $\lambda _5(T)=c_2$. The optimality condition, in the interior of the control’s set *W*, gives

8 $$\begin{aligned} \left\{ \begin{array}{l} \dfrac{\partial H}{ \partial {v}} = c_5+ 2c_6 v +c_9u- \lambda _1 M=0, \\ \\ \dfrac{\partial H}{ \partial {u}} = c_7+ 2c_8 u+c_9 v - \lambda _5 O=0, \end{array}\right. \end{aligned}$$

and, applying the bounds on *v* and *u* we get the optimal control characterization, recalling that $4c_6c_8-c_9^2> 0$

9 $$\begin{aligned} \left\{ \begin{array}{l} v^*= \min \left( v_{max}, \max \left( 0,\dfrac{(\lambda _1 M-c_5)2c_8-(\lambda _5 O-c_7)c_9}{4c_6c_8-c_9^2} \right) \right) , \\ \\ u^* = \min \left( u_{max}, \max \left( 0,\dfrac{(\lambda _5 O-c_7)2c_6-(\lambda _1 M-c_5)c_9}{4c_6c_8-c_9^2} \right) \right) . \end{array}\right. \end{aligned}$$

The optimality system is then given by equation (1) with $\alpha _0=\alpha _1=1$, with initial conditions $M(t=0) = M_0$, $U_2(t=0) = U_{20}$, $U_3(t=0) = U_{30}$, $U_4(t=0) = U_{40}$, $O(t=0) = O_{0}$ and the adjoint system (7) with the final time conditions $\lambda _1(T)=c_1$, $\lambda _2(T)=\lambda _3(T)=\lambda _4(T)=0$ and $\lambda _5(T)=c_2$, subject to (9). We solve it numerically in Sect. 5.

### Optimal control acting on monomers (CM)

In this subsection we will consider the case of a control *v*(*t*) which acts on the monomers’s population *M*(*t*) in the model (1) with $\alpha _0=1$ and $\alpha _1=0$. We will introduce a given objective functional in order to minimize both the populations *M*(*t*) and *O*(*t*) at the end of the treatment ($t=T$ with weights $c_1, c_2$) and over the treatment (in [0, *T*] with weights $c_3, c_4$), the cost of the therapy over the treatment (with weight $c_5$) and its side effects over the treatment (with weights $c_6$), i.e.

10 $$\begin{aligned} J(v) = c_1 M(T) +c_2 O(T)+ \int _0^T \left[ c_3 M(t)+c_4 O(t) +c_5 v(t)+c_6 v(t)^2 \right] dt, \end{aligned}$$

where $c_i$, with $i=1,2,...,6$, are positive constants. The state variables *M*(*t*), $U_2(t)$, $U_3(t)$, $U_4(t)$ and *O*(*t*) and the control *v*(*t*) are subject to (1) with $\alpha _0=1$ and $\alpha _1=0$, (with $u(t)=0$) and to the i.c. of system (1), i.e. $M_0$, $U_{20}$, $U_{30}$, $U_{40}$, $O_{0}$. Our aim is to find an optimal control $v^*$ such that

11 $$\begin{aligned} J(v^*) = \min _{v \in V} J(v), \end{aligned}$$

where $V= \lbrace v(t) \in L^{\infty }( \left[ 0,T \right] ) \quad | \quad 0 \le v(t) \le v_{max} \rbrace$. $v_{max}$ is the maximum rate of the therapy *v*. In order to find the optimal control $v^*$ we introduce the five adjoint variables, respectively $\lambda _i$, $i=1,2,...5$ and form the Hamiltonian:

12 $$\begin{aligned} H = c_3 M(t)+c_4 O(t) +c_5 v(t)+c_6 v(t)^2 + \lambda _1 \dfrac{dM}{dt}+ \lambda _2 \dfrac{dU_2}{dt} + \lambda _3 \dfrac{dU_3}{dt}+ \lambda _4 \dfrac{dU_4}{dt}+ \lambda _5 {\dfrac{dO}{dt}}, \end{aligned}$$

where $\dfrac{dM}{dt}$, $\dfrac{dU_2}{dt}$, $\dfrac{dU_3}{dt}$, $\dfrac{dU_4}{dt}$ and $\dfrac{dO}{dt}$ are the RHS of the ODE system (1) with $\alpha _0=1$ and $\alpha _1=0$. The adjoint ODE system with transversality conditions is given in the Appendix, see (25). From the optimality condition in the interior of *V* we get

13 $$\begin{aligned} \dfrac{\partial H}{ \partial v} = c_5+ 2c_6 v- \lambda _1 M=0, \end{aligned}$$

and, by the bounds on *v* we get the optimal control characterization

14 $$\begin{aligned} v^* = \min \left( v_{max}, \max \left( 0,\dfrac{\lambda _1 M-c_5}{2c_6} \right) \right) . \end{aligned}$$

The optimality system is then given by equation (1) with $\alpha _0=1$ and $\alpha _1=0$, with initial conditions $M_0$, $U_{20}$, $U_{30}$, $U_{40}$, $O_{0}$ and the adjoint system (25) with the final time conditions $\lambda _1(T)=c_1$, $\lambda _2(T)=\lambda _3(T)=\lambda _4(T)=0$ and $\lambda _5(T)=c_2$, subject to (13). We solve it numerically in Sect. 5.

### Optimal control acting on oligomers (CO)

Here we will consider the case of a control *u*(*t*) which acts on the oligomers’s population *O*(*t*) in the model (1) with $\alpha _0=0$ and $\alpha _1=1$. As before, we will introduce a given objective functional in order to minimize both the populations *M*(*t*) and *O*(*t*) at the end of the treatment ($t=T$ with weights $c_1, c_2$) and over the treatment (in [0, *T*] with weights $c_3, c_4$), the cost of the therapy over the treatment (with weight $c_7$) and its side effects over the treatment (with weights $c_8$), i.e.

15 $$\begin{aligned} J(u) = c_1 M(T) +c_2 O(T)+ \int _0^T \left[ c_3 M(t)+c_4 O(t) +c_7 u(t)+c_8 u(t)^2 \right] dt, \end{aligned}$$

where $c_1, c_2, c_3,c_4,c_7,c_8$ are positive constants. The state variables *M*(*t*), $U_2(t)$, $U_3(t)$, $U_4(t)$ and *O*(*t*) and the control *v*(*t*) are subject to (1) with $\alpha _0=0$ and $\alpha _1=1$, (with $u(t)=0$) and to the i.c. of system (1), i.e. $M_0$, $U_{20}$, $U_{30}$, $U_{40}$, $O_{0}$. Our goal is to find an optimal control $u^*$ such that

16 $$\begin{aligned} J(u^*) = \min _{u \in U} J(u), \end{aligned}$$

where $U= \lbrace u(t) \in L^{\infty }( \left[ 0,T \right] ) \quad | \quad 0 \le u(t) \le u_{max} \rbrace$. $u_{max}$ is the maximum rate of the therapy u . In order to find the optimal control $u^*$ we introduce the five adjoint variables, respectively $\lambda _i$, $i=1,2,...5$ and form the Hamiltonian:

17 $$\begin{aligned} H = c_3 M(t)+c_4 O(t) +c_7 u(t)+c_8 u(t)^2 + \lambda _1 {\dfrac{dM}{dt}}+ \lambda _2 \dfrac{dU_2}{dt} + \lambda _3 \dfrac{dU_3}{dt}+ \lambda _4 \dfrac{dU_4}{dt}+ \lambda _5\dfrac{dO}{dt}, \end{aligned}$$

where ${\dfrac{dM}{dt}}$ $\dfrac{dU_2}{dt}$, $\dfrac{dU_3}{dt}$, $\dfrac{dU_4}{dt}$ and $\dfrac{dO}{dt}$, are the RHS of the ODE system (1) with $\alpha _0=0$ and $\alpha _1=1$. The adjoint ODE system with transversality conditions is given in the Appendix, see (26). From the optimality condition in the interior of the set *U* we get

18 $$\begin{aligned} \dfrac{\partial H}{ \partial u} = c_7+ 2c_8 u - \lambda _5 O=0, \end{aligned}$$

and, by the bounds on *u*, we get the optimal control characterization

19 $$\begin{aligned} u^* = \min \left( u_{max}, \max \left( 0,\dfrac{\lambda _5 O-c_7}{2c_8} \right) \right) . \end{aligned}$$

The optimality system is then given by equation (1) with $\alpha _0=0$ and $\alpha _1=1$, with initial conditions $M_0$, $U_{20}$, $U_{30}$, $U_{40}$, $O_{0}$ and the adjoint system (26) with the final time conditions $\lambda _1(T)=c_1$, $\lambda _2(T)=\lambda _3(T)=\lambda _4(T)=0$ and $\lambda _5(T)=c_2$, subject to (18). We solve it numerically in Sect. 5.

### Optimal control acting on both monomers and oligomers with a single treatment (CMOST)

Finally, we consider here the case of a single control *w*(*t*) which acts simultaneously on the monomers’s population *M*(*t*) and the oligomer’s population *O*(*t*), in the model (1) with $\alpha _0=\alpha _1=1$ and $u=v=w$. The objective functional *J*(*w*) is defined considering the populations *M*(*t*) and *O*(*t*) at the end of the treatment ($t=T$ with weights $c_1, c_2$) and over the treatment (in [0, *T*] with weights $c_3, c_4$), the cost of the therapy over the treatment (with weight $c_5$) and its side effects over the treatment (with weights $c_6$), i.e.

20 $$\begin{aligned} J(w) = c_1 M(T) +c_2 O(T)+ \int _0^T \left[ c_3 M(t)+c_4 O(t) +c_5 w(t)+c_6 w(t)^2 \right] dt, \end{aligned}$$

where $c_i$, $i=1,2,...,6$ are positive constants. The state variables *M*(*t*), $U_2(t)$, $U_3(t)$, $U_4(t)$ and *O*(*t*) and the control *w*(*t*) are subject to (1) with $\alpha _0=\alpha _1=1$ (with $u(t)=v(t)=w(t)$) and initial conditions $M_0$, $U_{20}$, $U_{30}$, $U_{40}$, $O_{0}$. We seek to find an optimal control $w^*$ such that

21 $$\begin{aligned} J(w^*) = \min _{w \in W} J(w), \end{aligned}$$

where $W= \lbrace w(t) \in L^{\infty }( \left[ 0,T \right] ) \quad | \quad 0 \le w(t) \le w_{max} \rbrace$. $w_{max}$ is the maximum rate of the therapy *w*. In order to find the optimal control $w^*$ we introduce the five adjoint variables, respectively $\lambda _i$, $i=1,2,...5$ and form the Hamiltonian:

22 $$\begin{aligned} H = c_3 M(t)+c_4 O(t) +c_5 w(t)+c_6 w(t)^2 + \lambda _1 \dfrac{dM}{dt}+ \lambda _2 \dfrac{dU_2}{dt} + \lambda _3 \dfrac{dU_3}{dt}+ \lambda _4 \dfrac{dU_4}{dt}+ \lambda _5 {\dfrac{dO}{dt}}, \end{aligned}$$

where $\dfrac{dM}{dt}$, $\dfrac{dU_2}{dt}$, $\dfrac{dU_3}{dt}$, $\dfrac{dU_4}{dt}$ and $\dfrac{dO}{dt}$ are the RHS of the ODE system (1) with $\alpha _0=\alpha _1=1$ and $u(t)=v(t)=w(t)$. The adjoint ODE system is given in the Appendix, see (27). From the optimality condition in the interior of *W* we get

23 $$\begin{aligned} \dfrac{\partial H}{ \partial w} = c_5+ 2c_6 w- \lambda _1 M-\lambda _5 O=0, \end{aligned}$$

and, by the bounds on *w*, we get the optimal control characterization

24 $$\begin{aligned} w^* = \min \left( w_{max}, \max \left( 0,\dfrac{\lambda _1 M+\lambda _5 O-c_5}{2c_6} \right) \right) . \end{aligned}$$

The optimality system is then given by equation (1) with $\alpha _0=\alpha _1=1$ and $u(t)=v(t)=w(t)$, with initial conditions $M_0$, $U_{20}$, $U_{30}$, $U_{40}$, $O_{0}$ and the adjoint system (27) with the final time conditions $\lambda _1(T)=c_1$, $\lambda _2(T)=\lambda _3(T)=\lambda _4(T)=0$ and $\lambda _5(T)=c_2$, subject to (23). We solve it numerically in Sect. 5.

## Numerical results

In this section we present numerical results related to the optimal control formulations introduced in the previous section. All the simulations were made in MATLAB. Next, we will present four different scenarios: results for the case when only one treatment is administered, i.e. for the degradation of monomers (CM) or oligomers (CO), respectively, a combined treatment for the degradation of both monomers and oligomers (CMO) and, last, the same treatment for both monomers and oligomers (CMOST), respectively. We will compare the four different strategies, i.e. CM, CO, CMO and CMOST, in terms of the trend of the populations of monomers, *M*, proto-oligomers of size 2, 3 and 4, here the sum $U_2+U_3+U_4$ was considered, and of oligomers, *O*, for the case without control, with maximum control and optimal control. Furthermore, we also present results of the optimal controls for the third case (CMO) where some key parameters of the model can assume different values, as well as the initial conditions of monomers or oligomers.

### Numerical setting and parameter values

The numerical method used to approximate the optimal piecewise continuous controls $v^*$, $u^*$ and $w^*$, which solves the optimal control problems introduced in (5), (11), (16) and (21) is the forward-backward sweep method introduced in Lenhart and Workman (2007). The forward-backward sweep method is an iterative scheme, an initial guess for the controls, $v_0$, $u_0$ and $w_0$, are chosen and corresponding solutions to the state and adjoint equations are computed, substituting this last values in formulas (9), (14), (19) and (24) a first approximation of the controls is obtained. The process is repeated until the obtained sequences which approximates *v*, *u* and *w* converge to within a given tolerance in $L^2$ norm.

In Table 2 are summarized the parameters related to model (1), together with the values used for the simulations. With the exception of few cases, i.e. the new parameters introduced in our model, the values were taken from literature, as specified in the last column of the Table 2.

**Table 2:** Parameters of model (1). $1M = 1 l \ (mol)^{-1}$, is the molar concentration unit

Parameter	Name	Value	Unit	Refs
*s*	Monomers intrinsic growth rate	Variable	$\hbox {s}^{-1}$	–
*K*	Monomers carrying capacity	Variable	$\hbox {M}^{-1}$	–
$r_i$, $i=2,3,4$	Polymerization rate	100	M^−1^ s^−1^	Andrade-Restrepo et al. (2020)
*b*	Depolymerization rate	$10^{-3}$	$\hbox {s}^{-1}$	Andrade-Restrepo et al. (2020)
$\beta$	Fragmentation rate	$10^{-4}$	$\hbox {s}^{-1}$	Andrade-Restrepo et al. (2020)
$\delta$	Degradation rate of monomers	$5 \times 10^{-4}$	$\hbox {s}^{-1}$	Andrade-Restrepo et al. (2020)
$a_{11}$	Concatenation rate	10	M^−1^ s^−1^	Bertsch et al. (2016); Murphy and Pallitto (2000)
$a_{22}$	Concatenation rate	2.5	M^−1^ s^−1^	Bertsch et al. (2016); Murphy and Pallitto (2000)
$a_{23}$	Concatenation rate	1.67	M^−1^ s^−1^	Bertsch et al. (2016); Murphy and Pallitto (2000)
$a_{24}$	Concatenation rate	1.25	M^−1^ s^−1^	Bertsch et al. (2016); Murphy and Pallitto (2000)
$a_{33}$	Concatenation rate	1.11	M^−1^ s^−1^	Bertsch et al. (2016); Murphy and Pallitto (2000)
$a_{34}$	Concatenation rate	0.83	M^−1^ s^−1^	Bertsch et al. (2016); Murphy and Pallitto (2000)
$a_{44}$	Concatenation rate	0.625	M^−1^ s^−1^	Bertsch et al. (2016); Murphy and Pallitto (2000)
*m*	Oligomers intraspecific competition rate	Variable	M^−1^ s^−1^	-

Furthermore, Table 3 introduces the initial conditions of the five populations in model (1) used in the simulations and Table 4 presents the weight parameters in the objective functions to be minimized, introduced in (4), (10), (15) and (20), respectively. If not otherwise specified the parameters will assume fixed values as in Tables 2, 3 and 4.

**Table 3:** Initial concentration of the populations of model (1). $1M = 1 l \ (mol)^{-1}$, is the molar concentration unit

Initial conditions	Name	Value	Unit	
*M*(0)	Initial concentration of monomers	$10^{-3}$	$\hbox {M}^{-1}$	
$U_2(0)$	Initial concentration of proto-oligomers of length 2	0	$\hbox {M}^{-1}$	
$U_3(0)$	Initial concentration of proto-oligomers of length 3	0	$\hbox {M}^{-1}$	
$U_4(0)$	Initial concentration of proto-oligomers of length 4	0	$\hbox {M}^{-1}$	
*O*(0)	Initial concentration of oligomers	0	$\hbox {M}^{-1}$	-

**Table 4:** Weights values related to the objective functionals (4), (10), (15) and (20)

Parameters	Value
$c_i, i=1,2,3,4$	1
$c_i, i=5,7,9$	$10^{-5}$
$c_i, i=6,8$	2

### Comparison between treatments for monomers and/or oligomers

In this subsection we first compare the optimal control results for CM, CO, CMO and CMOST as well as the state variables associated with them.

**Fig. 2:**
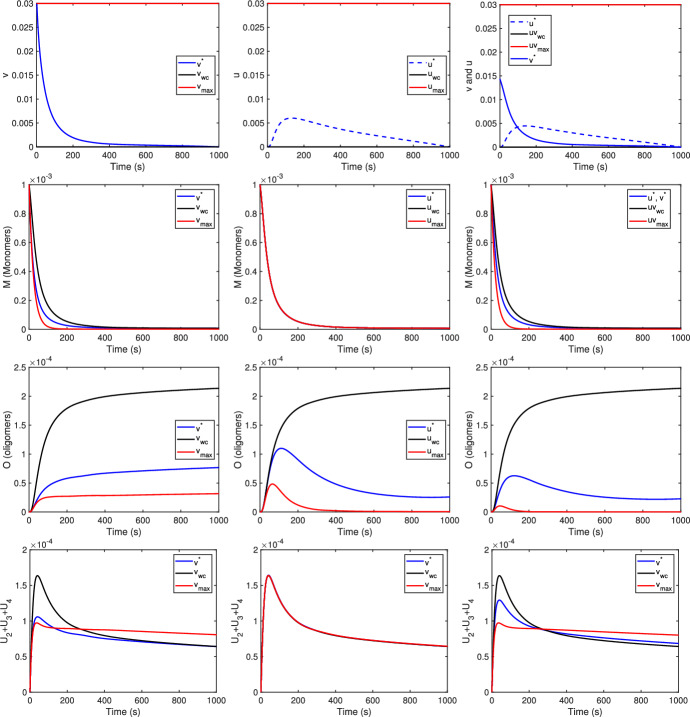
First row: optimal controls. From second to fourth rows: state variables *M*, *O*, and $U_2+U_3+U_4$, respectively, of model (1) for the optimal control case, without control and with maximum control, respectively. $v^*$ (continuous blue line) and $u^*$ (dashed blue line) are the results obtained from the optimal control formulation; $v_{wc}$ and $u_{wc}$ (black lines) refers to the case without control while $v_{max}$ and $u_{max}$ (red lines) to the case where the maximum dose is administered. The results on the first column refer to the case CM (control only on monomers), the second row to the case CO (control only on oligomers) and the third column to the case CMO (control both on monomers and oligomers). The parameter values of the model are as in Tables 2, 3 and 4. $s=m=10^{-4}$, $K=1$

In the first two columns of Fig. 2, the results for CM and CO, respectively, are compared. In the first column, the control acts only on monomers, whereas in the second it acts only on oligomers. In particular, the optimal controls $v^*$ and $u^*$ for the two cases are compared, as well as the state variables corresponding to the optimal controls, and the state variables of the model (1) for the case when the maximum dose of treatment is administered. Moreover, the aforementioned results can be compared with those where no treatment is considered. From the first row of Fig. 2 one can see that the shape of the two optimal controls are different. If the treatment acts only on monomers, then the optimal control assumes its maximum in the first days of treatment and then decreases until reaching zero at the final time of treatment ($T=1000$ s); If the treatment acts on oligomers instead, the optimal control starts from zero at the beginning of the treatment, increases until reaching a maximum value 0.006 at time $t=113$ s, and then slowly decreases until the final time $T=1000$ s.

Let us now compare the curves describing the evolution of the state variables $M, U_j, j=2,3,4, O$ of model (1) when the optimal control $v^*$ or $u^*$ is chosen. First, acting on monomers has an impact on all the populations of the model. We observe that the treatment decreases the monomers *M* from *M*(0) to $M(1000)=7.1168e-06$, maintaining at the same time the oligomers at a low level ($O(1000)=7.6725e-05$, lower than $O(1000)=2.1373e-04$ without control) and substantially decreasing the proto-oligomer populations of size 2, 3 and 4.

Acting only on the oligomers instead has a significant impact only on the oligomer population itself. After a first increasing phase, the oligomer population decreases to $O(1000)=2.5942e-05$. Notice that this value is smaller than the one obtained controlling only the monomers but it is close to the value obtained treating the monomers with the maximum treatment, $O(1000)=3.1621e-05$.

Second, comparing the effect of the treatments on monomers and oligomers at maximal dose, the oligomer treatment seems more efficient, as the red curves in the second column of Fig. 2 are below or equal to the red ones of the first column.

Furthermore, in the last column of Fig. 2, a joint control on both *M* and *O* is considered. In this case, the optimal controls $v^*$ and $u^*$ (qualitatively) maintain the same trend; however, the doses for both treatments are lower compared with the case when only one treatment is considered (columns one and two). Acting both on *M* and *O* has an impact on all the populations of the model (1), and it is a combination of the “best” results from only one treatment.

**Fig. 3:**
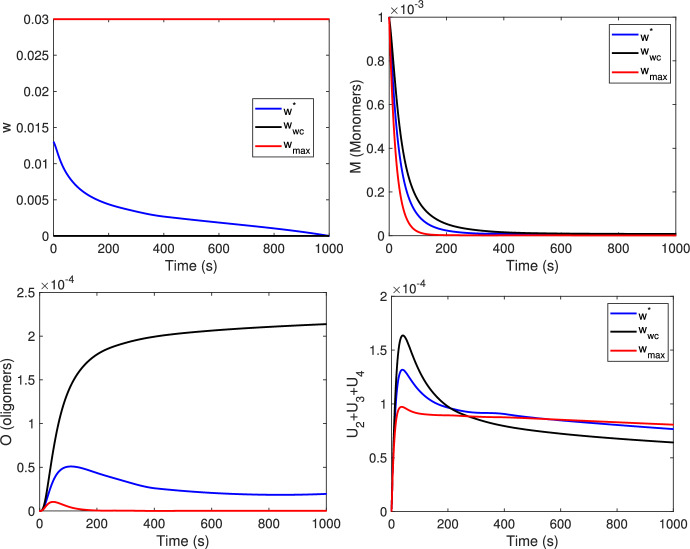
First row: optimal control and state variable *M*; second row: state variables *O*, and $U_2+U_3+U_4$, for the optimal control case, without control and with maximum control, respectively. $w^*$ is the result obtained from the optimal control formulation; $w_{wc}$ (black line) refers to the case without control while $w_{max}$ (red line) to the case where the maximum dose is administered. The results of the first column refers to the case CMO (control both monomers and oligomers) with the same treatment. The parameter values of the model are as in Tables 2, 3 and 4. $s=m=10^{-4}$, $K=1$

In Figure 3 the results for the case where the same treatment is used for both the degradation of the monomers and oligomers (CMOST) are plotted. The optimal control $w^*$, compared with $v^*$ and $u^*$ obtained in the previously considered scenarios, starts at a maximum value at the beginning of the treatment, which is in any case lower than in the cases seen before, and then decreases gradually until $T=1000$ ${s}$, which corresponds to the end of the treatment. Hence, in the first days of treatment $w^*$ behaves as $v^*$ and in the last days as $u^*$. Comparing the state variables associated with the optimal control $w^*$ with those corresponding to $v^*$ or $u^*$, the monomer curve $M[w^*]$ behaves qualitatively as $M[v^*]$, whereas $O[w^*]$ behaves similarly to $O[u^*]$.

Table 5 summarizes the values of the objective functional evaluated at the optimal control value, at the maximum treatment value and for the case without treatment, for CM, CO, CMO, CMOST. Moreover, the doses of the treatments for the results reported in Figs. 2 and 3 are listed. In Table 5$u_{max}$, $v_{max}$ and $w_{max}$ are the maximum rate of treatment to be administered for oligomers and monomers degradation, respectively. $J(v^*)$, $J(v_{wc})$ and $J(v_{max})$ are the values of the objective functional evaluated at the optimal value of the control, when no control is considered and when the maximum dose is considered, respectively, for the monomers, $J(u^*)$, $J(u_{wc})$ and $J(u_{max})$ for the oligomers, $J(v^*,u^*)$, $J(v_{wc}, u_{wc})$ and $J(v_{max}, u_{max},)$ when both treatments are applied and $J(w^*)$, $J(w_{wc})$ and $J(w_{max})$ when the same treatment is applied to both monomers and oligomers. Then $dose(v^*)$ and $dose(v_{max})$ are the doses, for the monomers treatment, for the optimal control case and the case with maximum control, respectively, $dose(u^*)$ and $dose(u_{max})$ same as before but for oligomers, while $dose(w^*)$ and $dose(w_{max})$ same as before but for both *M* and *O*. For a generic treatment *p* we compute

$$dose(p) = \int _0^T p(t)dt.$$

**Table 5:** Values of the objective functional and the doses of the treatments for the results reported in Figs. 2 and 3. The case CM: control only monomers, the case CO: control only oligomers, and, third case CMO: control both monomers and oligomers

CM	$J(v^*) = 0.15229$		$dose(v^*)= 2.042$
	$J(v_{max}) = 1.850$		$dose(v_{max})= 29.97$
	$J(v_{wc}) = 0.2529$		$dose(v_{wc}) = 0$
CO	$J(u^*) = 0.1381$	$dose(u^*) = 2.914$	
	$J(u_{max}) = 1.871$	$dose(u_{max}) = 29.97$	
	$J(u_{wc}) = 0.2529$	$dose(u_{wc}) = 0$	
CMO	$J( v^*,u^*) = 0.1148$	$dose(u^*) = 2.306$	$dose(v^*)=1.439$
	$J(v_{max}, u_{max}) = 3.619$	$dose(u_{max}) = 29.97$	$dose(v_{max}) = 29.97$
	$J(v_{wc}, v_{wc}) = 0.2529$	$dose(u_{wc}) = 0$	$dose(v_{wc}) = 0$
CMOST	$J(w^*) = 0.1040$	$dose(w^*) = 2.958$	
	$J(w_{max}) = 1.823$	$dose(w_{max}) = 29.97$	
	$J(w_{wc}) = 0.2529$	$dose(w_{wc}) = 0$	

### Investigation on parameter values and initial conditions

In this subsection we investigate how the optimal control related to CMO changes when some parameters of the model or of the objective functions (see (5), (11), (16) and (21)) changes, as well as when the initial conditions of monomers and oligomers assume different values. From now on we will present numerical results related to the third case, i.e. CMO, since CM, CO and CMOST can be seen as particular cases of it.

**Fig. 4:**
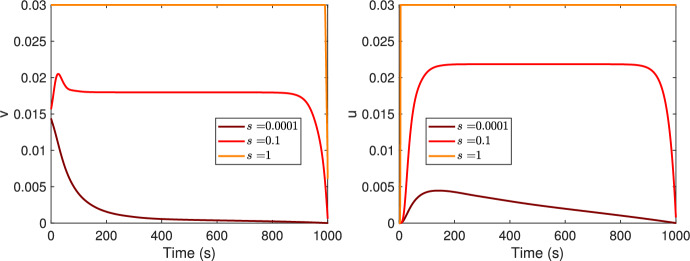
Optimal controls, $v^*$ (first column) and $u^*$ (second column), for three different values of the growth rate of the monomers, *s*. Increasing values of *s* as in legend

As can be seen in Table 2, three parameters, i.e. the monomers intrinsic growth rate, *s*, the monomers carrying capacity *K* and the oligomers intraspecific competition rate *m* are assumed variable. In fact, to best of our knowledge no data is present in literature about their values. In order to understand how different values of *s*, *K* or *m* impact on the output of the CMO optimal control formulation, numerical test were conducted. For the parameter values as in Tables 2, 3 and 4, with *K* or *m* assuming four different values in $\left[ 10^{-4},10^3 \right]$ or $\left[ 0,1 \right]$, respectively, the optimal controls $v^*$ and $u^*$ are the same as in Fig. 2 (first row, last column), results not reported here. While for three different values of *s*, i.e. $s=10^{-4}$, $s=0.1$ or $s=1$, three qualitative different optimal controls $v^*$ and $u^*$ are obtained (Fig. 4). For $s=10^{-4}$ the results are the same as in Fig. 2 (first row, last column). For $s=0.1$, meaning increasing the growth rate of the monomers, the control on the monomers starts with an initial peak to then remain constant at 0.018 from 120 s for almost all the treatment duration, before starting to drop at 800 s until to reach value zero for $t=1000$ s. On the other side, for $u^*$, the optimal treatment starts at a zero value and ends at zero, but for 172 $s< t < 853$ *s* it maintains the constant value 0.0218; note that this value is higher than the one corresponding to $v^*$ (0.018). For $s=1$, when the growth rate of monomers is high, both the treatments must be administered at their higher dose for all the duration of the treatment with the sole difference that $v^*$ starts from the maximum value 0.03 to end at zero at $t=1000$ s, while $u^*$ starts at zero and ends at the maximum value.

**Fig. 5:**
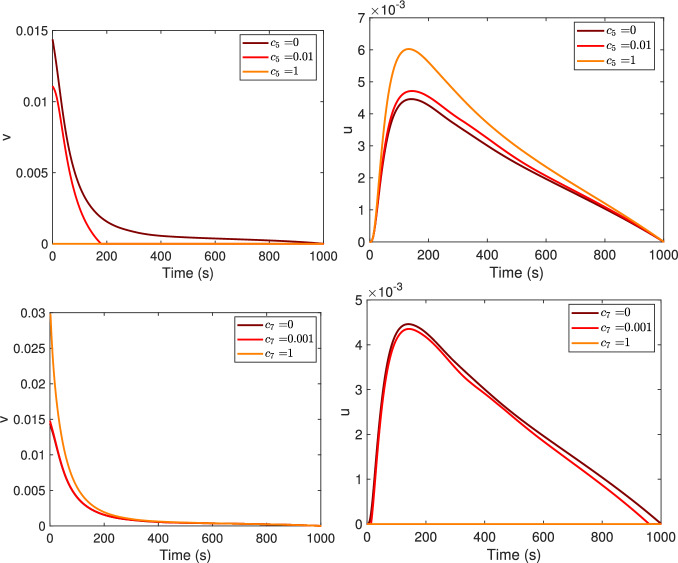
Optimal controls, $v^*$ (first column) and $u^*$ (second column), for three different values of the weights $c_5$ and $c_7$. Increasing values of $c_5$ and $c_7$ as in legend

In Fig. 5, the optimal controls $v^*$ and $u^*$ are compared for three different choices of the weights $c_5$ and $c_7$ in (5). Recall that $c_5$ and $c_7$ correspond to the effective cost of the treatment of the monomer and oligomer populations, respectively. Choosing a low value for $c_5$ results in a higher $v^*$ optimal control curve and a lower $u^*$ curve. For $c_5=1$, $v^*$ is constantly zero, while $u^*$ assumes the biggest dose, compared to the other lower $c_5$ values. A similar result is obtained for $c_7$, the cost of the treatment for oligomers, with the role of $v^*$ and $u^*$ being interchanged.

**Fig. 6:**
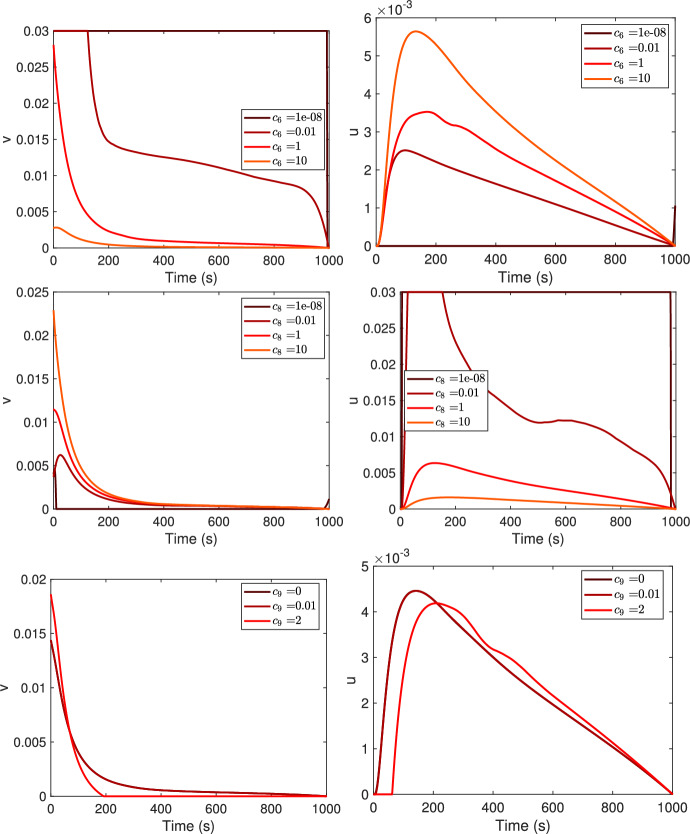
Optimal controls, $v^*$ (first column) and $u^*$ (second column), for four different values of the weights $c_6$, $c_8$ and $c_9$. Increasing values of $c_6$, $c_8$ and $c_9$ as in legend. Notice that the curves for $c_9 =0$ and $c_9=0.01$ overlap

In Fig. 6, the optimal controls $v^*$ and $u^*$ for four different values of the weights $c_6$, $c_8$ and $c_9$ in the objective functional (5) are compared. Recall that $c_6$, $c_8$, and $c_9$ correspond to the impact of the side effects for the treatment on the monomer, on the oligomer or for the combination of the two treatments, respectively. The higher $c_6$ is, the lower $v^*$ is and the higher $u^*$ is; conversely, the higher $c_8$ is, the higher $v^*$ is and the lower $u^*$ is. A similar pattern is also seen when the parameter $c_9$ changes, provided it verifies the convexity assumption $4c_6 c_8 - c_9^2 > 0$, see Fig. 6, where we chose the values $c_9=0$, $c_9 = 0.01$ and $c_9=2$.

**Fig. 7:**
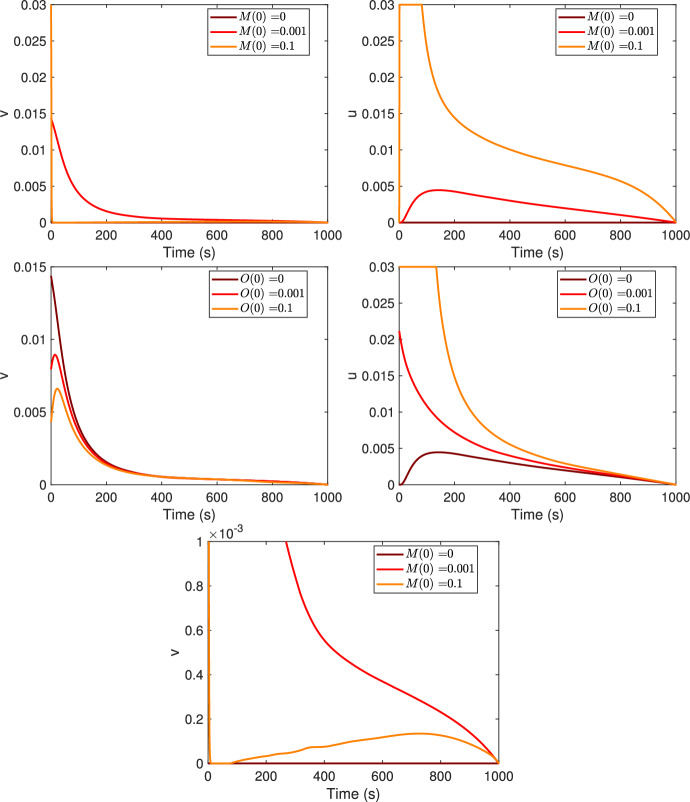
Optimal controls, $v^*$ (first column) and $u^*$ (second column), for three different values of initial conditions. First row, the initial condition of monomers is variable while the initial conditions for the other populations are fixed as in Table 3. Second row, the initial condition for the oligomers is variable, while it is as in Table 3 for the other populations. Third row, a zoom of the first panel. Increasing values of *M*(0) or *O*(0) as in legend

In Fig. 7 are represented the optimal control strategies for the monomers and oligomers treatment for three different i.c. of monomers or oligomers, e.g., $M(0)=0$, $M(0)=10^{-3}$ and $M(0)=0.1$, first row, the same values are considered for the oligomers, assuming fixed the other i.c. values, second row. Comparing the obtained results one can see that higher *M*(0) or *O*(0) are, higher the treatment of the oligomers must be.

### Brief summary of the numerical results

The numerical study introduced in this section has two main purposes. On one hand we wanted to compare four different treatment strategies, by computing the corresponding numerical optimal controls. On the other hand, we have analyzed the behaviour of the optimal controls with respect to some key parameter values and different initial conditions of the considered populations.

For the four different treatment strategies we have learned that a combined treatment for the degradation of both monomers and oligomers is more efficient in terms of depleting all the populations of the model, differently that happens where only one treatment at the time is considered. In fact acting only on oligomers has a direct impact only on the oligomers population itself.

Regarding the study of some key parameter values, we have learned that higher the intrinsic growth rate of monomers is, a higher dose of treatment is need. Moreover, the optimal control that acts on monomers starts at the maximum value, at the beginning of the treatment, to then decrease at the end, while the optimal control that acts on the oligomers starts at its lowest value to then increase and eventually decrease again at the end of the treatment. Moreover, for the analysis of the impact of the initial condition of monomers and oligomers on the optimal control output, one can see that the presence of the oligomers, at high or low values at the beginning of the treatment, will demand higher treatment doses for both treatments, differently that for the monomer’s initial conditions where the optimal control is more relevant only for monomer’s population.

## Conclusions and future research directions

The mathematical models are often used as complementary tools to better understand biological systems. Recently, ODE and PDE models were used to describe the interaction of the main actors of the Alzheimer’s disease, Andrade-Restrepo et al. (2021); Bertsch et al. (2018, 2016), Andrade-Restrepo et al. (2020); Achdou et al. (2013) with a particular focus on the Amyloid Cascade Hypothesis (ACH), which is one of the most widely studied and debated, Hardy and Higgins (1992).

Here a mathematical model for the biochemical interactions between monomers, proto-oligomers, and oligomers of A$\beta$ amyloids, was developed by assuming that the interactions between the populations of the ordinary differential equation (ODE) model are: polymerization, depolymerization, fragmentation, degradation and concatenation. Moreover, we have also introduced two different controls, one on the monomers population and a second one on the oligomers. For the introduced model four different optimal control formulations were studied. First, we have assumed that only one in between the monomers and oligomers population is degraded, CM and CO, respectively. Secondly, we have assumed that both the populations can be degraded, by the same treatment or a different one, CMOST and CMO, respectively.

Comparing the numerical results for the four cases one can conclude that acting only on monomers or only on oligomers leads to different effects on the five populations of the model. Degrading only the monomers, with an optimal control, will have a chain effect on all the populations of the model, by decreasing them at the end of the treatment if compared to the case where no treatment was administered, while, degrading only the oligomers, again with an optimal control, will affect only the population for which the treatment is considered. Furthermore, if both the monomers and oligomers are subject to two optimal control treatments simultaneously, the two optimal controls assume the same shape as for the first two cases but with lower dosed, while all the populations of the model decrease at the end of the treatment.

If the same treatment is administered for both monomers and oligomers, the numerical evidence shows that only one treatment is more advantageous in terms of the treatment doses needed, for the optimal case. Moreover, the objective functional that has been minimized assumes the minimum value if compared with the other three cases.

Modeling Alzheimer’s disease is a growing research area, in particular there are several future research directions one might want to explore and extend. On one hand, the study of a PDE model that describes the dynamics and aggregation of A$\beta$-amyloids in a small neighbourhood of a few neurons, Bertsch et al. (2018), might give interesting insights on the disease. On the other hand, implement optimal control formulations also for models describing a more advanced stage of the disease, where the plaques are already formed, Ficiarà et al. (2023), could help in finding the best treatment strategy. Moreover, would be interesting to combine the above mentioned studies with clinical data related to *in vitro* (Sancesario et al. 2018) or *in vivo* (Cummings et al. 2021) treatments.

## Data Availability

The manuscript has no associated data.

## Appendix

We provide here some auxiliary equations for the study of the optimal controls of the Sect. 4. In the case of optimal control acting on the monomers (CM), the adjoint ODE system reads

25 $$\begin{aligned} \dfrac{d \lambda _1}{dt}= & - \dfrac{\partial H}{ \partial M} = - \biggl [ c_3 + \lambda _1 (s- \delta -v(t)) + M \left[ \dfrac{-2 \lambda _1 s}{K} -2 a_{11} \lambda _1 + 2 \lambda _2 a_{11} \right] + \biggr . \nonumber \\ & \biggl . + r_2 U_2(- \lambda _1 - \lambda _2 + \lambda _3) + r_3 U_3 (- \lambda _1 - \lambda _3+ \lambda _4) +r_4 U_4 (-\lambda _1 - \lambda _4 + \lambda _5) \biggr ] \nonumber \\ \dfrac{d \lambda _2}{dt}= & - \dfrac{\partial H}{ \partial U_2} = - \left[ \beta (- \lambda _2 + 2 \lambda _1) + r_2 M (- \lambda _1 - \lambda _2 + \lambda _3) + 2 a_{22} U_2 (-\lambda _2 + \lambda _4)+ \right. \nonumber \\ & \left. +a_{23} U_3 (- \lambda _2 - \lambda _3 + \lambda _5) + a_{24} U_4 (- \lambda _2 - \lambda _4 + \lambda _5) \right] \nonumber \\ \dfrac{d \lambda _3}{dt}= & - \dfrac{\partial H}{ \partial U_3} = - \left[ (b+2 \beta ) ( \lambda _1 + \lambda _2 - \lambda _3) +r_3 M (- \lambda _1 - \lambda _3 + \lambda _4)+ \right. \nonumber \\ & \left. +a_{23} U_2 (- \lambda _2 - \lambda _3 + \lambda _5) +2 a_{33} U_3 (-\lambda _3+ \lambda _5) +a_{34} U_4 (- \lambda _3 - \lambda _4 + \lambda _5) \right] \nonumber \\ \dfrac{d \lambda _4}{dt}= & - \dfrac{\partial H}{ \partial U_4} = -\left[ (b+2 \beta ) (\lambda _1 + \lambda _3) + 2 \beta \lambda _2 -(b+3 \beta ) \lambda _4+ r_4 M (-\lambda _1 - \lambda _4 + \lambda _5)+ \right. \nonumber \\ & \left. +a_{24} U_2 (-\lambda _2 - \lambda _4 + \lambda _5) +a_{34} U_3 (-\lambda _3 - \lambda _4 + \lambda _5) +2 a_{44} U_4 (-\lambda _4+ \lambda _5) \right] \nonumber \\ \dfrac{d \lambda _5}{dt}= & - \dfrac{\partial H}{ \partial O} = -(c_4-2 M \lambda _5 O), \end{aligned}$$

with transversality conditions $\lambda _1(T)=c_1$, $\lambda _2(T)=\lambda _3(T)=\lambda _4(T)=0$ and $\lambda _5(T)=c_2.$

In the case of optimal control acting on the oligomers (CO), the adjoint ODE system reads

26 $$\begin{aligned} \dfrac{d \lambda _1}{dt}= & - \dfrac{\partial H}{ \partial M} = - \biggl [ c_3 + \lambda _1 (s- \delta ) + M \left[ \dfrac{-2 \lambda _1 s}{K} -2 a_{11} \lambda _1 + 2 \lambda _2 a_{11} \right] \biggr . \nonumber \\ & \biggl . + r_2 U_2(- \lambda _1 - \lambda _2 + \lambda _3) + r_3 U_3 (- \lambda _1 - \lambda _3+ \lambda _4) +r_4 U_4 (-\lambda _1 - \lambda _4 + \lambda _5) \biggr ] \nonumber \\ \dfrac{d \lambda _2}{dt}= & - \dfrac{\partial H}{ \partial U_2} = - \left[ \beta (- \lambda _2 + 2 \lambda _1) + r_2 M (- \lambda _1 - \lambda _2 + \lambda _3) + 2 a_{22} U_2 (-\lambda _2 + \lambda _4) \right. \nonumber \\ & \left. +a_{23} U_3 (- \lambda _2 - \lambda _3 + \lambda _5) + a_{24} U_4 (- \lambda _2 - \lambda _4 + \lambda _5) \right] \nonumber \\ \dfrac{d \lambda _3}{dt}= & - \dfrac{\partial H}{ \partial U_3} = - \left[ (b+2 \beta ) (\lambda _1 + \lambda _2 - \lambda _3) +r_3 M (- \lambda _1 - \lambda _3 + \lambda _4) \right. \nonumber \\ & \left. +a_{23} U_2 (- \lambda _2 - \lambda _3 + \lambda _5) +2 a_{33} U_3 (-\lambda _3+ \lambda _5) +a_{34} U_4 (- \lambda _3 - \lambda _4 + \lambda _5) \right] \nonumber \\ \dfrac{d \lambda _4}{dt}= & - \dfrac{\partial H}{ \partial U_4} = -\left[ (b+2 \beta ) (\lambda _1 + \lambda _3) + 2 \beta \lambda _2 -(b+3 \beta ) \lambda _4+ r_4 M (-\lambda _1 - \lambda _4 + \lambda _5) \right. \nonumber \\ & \left. +a_{24} U_2 (-\lambda _2 - \lambda _4 + \lambda _5) +a_{34} U_3 (-\lambda _3 - \lambda _4 + \lambda _5) +2 a_{44} U_4 (-\lambda _4+ \lambda _5) \right] \nonumber \\ \dfrac{d \lambda _5}{dt}= & - \dfrac{\partial H}{ \partial O} = -(c_4 + \lambda _5 (- 2 M O - u(t)) ), \end{aligned}$$

with transversality conditions $\lambda _1(T)=c_1$, $\lambda _2(T)=\lambda _3(T)=\lambda _4(T)=0$ and $\lambda _5(T)=c_2.$

In the case of optimal control acting on both monomers and oligomers (CMOST), the adjoint ODE system reads

27 $$\begin{aligned} \dfrac{d \lambda _1}{dt}= & - \dfrac{\partial H}{ \partial M} = - \biggl [ c_3 + \lambda _1 (s- \delta - w(t)) + M \left[ \dfrac{-2 \lambda _1 s}{K} -2 a_{11} \lambda _1 + 2 \lambda _2 a_{11} \right] \biggr . \nonumber \\ & \biggl . + r_2 U_2(- \lambda _1 - \lambda _2 + \lambda _3) + r_3 U_3 (- \lambda _1 - \lambda _3+ \lambda _4) +r_4 U_4 (-\lambda _1 - \lambda _4 + \lambda _5) \biggr ] \nonumber \\ \dfrac{d \lambda _2}{dt}= & - \dfrac{\partial H}{ \partial U_2} = - \left[ \beta (- \lambda _2 + 2 \lambda _1) + r_2 M (- \lambda _1 - \lambda _2 + \lambda _3) + 2 a_{22} U_2 (-\lambda _2 + \lambda _4) \right. \nonumber \\ & \left. +a_{23} U_3 (- \lambda _2 - \lambda _3 + \lambda _5) + a_{24} U_4 (- \lambda _2 - \lambda _4 + \lambda _5) \right] \nonumber \\ \dfrac{d \lambda _3}{dt}= & - \dfrac{\partial H}{ \partial U_3} = - \left[ (b+2 \beta ) ( \lambda _1 + \lambda _2 - \lambda _3) +r_3 M (- \lambda _1 - \lambda _3 + \lambda _4) \right. \nonumber \\ & \left. +a_{23} U_2 (- \lambda _2 - \lambda _3 + \lambda _5) +2 a_{33} U_3 (-\lambda _3+ \lambda _5) +a_{34} U_4 (- \lambda _3 - \lambda _4 + \lambda _5) \right] \nonumber \\ \dfrac{d \lambda _4}{dt}= & - \dfrac{\partial H}{ \partial U_4} = -\left[ (b+2 \beta ) (\lambda _1 + \lambda _3) + 2 \beta \lambda _2 -(b+3 \beta ) \lambda _4+ r_4 M (-\lambda _1 - \lambda _4 + \lambda _5) \right. \nonumber \\ & \left. +a_{24} U_2 (-\lambda _2 - \lambda _4 + \lambda _5) +a_{34} U_3 (-\lambda _3 - \lambda _4 + \lambda _5) +2 a_{44} U_4 (-\lambda _4+ \lambda _5) \right] \nonumber \\ \dfrac{d \lambda _5}{dt}= & - \dfrac{\partial H}{ \partial O} = -(c_4 + \lambda _5 (- 2 M O - w(t)) ), \end{aligned}$$

with transversality conditions $\lambda _1(T)=c_1$, $\lambda _2(T)=\lambda _3(T)=\lambda _4(T)=0$ and $\lambda _5(T)=c_2.$
